# The Mre11-Rad50-Xrs2 Complex Is Required for Yeast DNA Postreplication Repair

**DOI:** 10.1371/journal.pone.0109292

**Published:** 2014-10-24

**Authors:** Lindsay G. Ball, Michelle D. Hanna, Amanda D. Lambrecht, Bryan A. Mitchell, Barry Ziola, Jennifer A. Cobb, Wei Xiao

**Affiliations:** 1 Department of Microbiology and Immunology, University of Saskatchewan, Saskatoon, SK, Canada; 2 Department of Biochemistry and Molecular Biology, University of Calgary, Calgary, AB, Canada; 3 Department of Pathology and Laboratory Medicine, University of Saskatchewan, Saskatoon, SK, Canada; 4 College of Life Sciences, Capital Normal University, Beijing, China; Saint Louis University, United States of America

## Abstract

Yeast DNA postreplication repair (PRR) bypasses replication-blocking lesions to prevent damage-induced cell death. PRR employs two different mechanisms to bypass damaged DNA, namely translesion synthesis (TLS) and error-free PRR, which are regulated via sequential ubiquitination of proliferating cell nuclear antigen (PCNA). We previously demonstrated that error-free PRR utilizes homologous recombination to facilitate template switching. To our surprise, genes encoding the Mre11-Rad50-Xrs2 (MRX) complex, which are also required for homologous recombination, are epistatic to TLS mutations. Further genetic analyses indicated that two other nucleases involved in double-strand end resection, Sae2 and Exo1, are also variably required for efficient lesion bypass. The involvement of the above genes in TLS and/or error-free PRR could be distinguished by the mutagenesis assay and their differential effects on PCNA ubiquitination. Consistent with the observation that the MRX complex is required for both branches of PRR, the MRX complex was found to physically interact with Rad18 *in vivo*. In light of the distinct and overlapping activities of the above nucleases in the resection of double-strand breaks, we propose that the interplay between distinct single-strand nucleases dictate the preference between TLS and error-free PRR for lesion bypass.

## Introduction

In order to maintain genomic integrity, living organisms have developed a set of highly conserved mechanisms to deal with spontaneous and induced DNA damage. DNA lesions that result in stalled replication apparatus are among the most dangerous and result in genomic instability, a well-known hallmark of cancer. DNA repair and replication checkpoints act to prevent the collapse of blocked replication apparatus, while homologous recombination (HR) acts to rescue double-strand breaks (DSBs) induced by collapsed replication forks [1]. To prevent detrimental outcomes, the budding yeast *Saccharomyces cerevisiae RAD6* DNA postreplication repair (PRR) epistasis group functions to bypass replication blocks [2]. Rad6 is known to have diverse functions outside of PRR, while Rad18 functions in a stable complex with Rad6 to monoubiquitinate proliferating cell nuclear antigen (PCNA). PCNA is encoded by the essential gene *POL30* in budding yeast and is a DNA polymerase sliding clamp. Current evidence suggests that upon exposure to DNA damage, PCNA is monoubiquitinated at the K164 residue [3] and that this monoubiquitination promotes translesion DNA synthesis (TLS). The TLS pathway is represented by *REV3* and *REV7*, which encode the catalytic and regulatory subunits of DNA polymerase ξ (Polξ) respectively, and *REV1*; inactivation of any one of the above genes results in a severely compromised induction of mutagenesis after DNA damage treatment and a reduction in spontaneous mutagenesis [4].

Monoubiquitinated PCNA can be further polyubiquitinated by Mms2-Ubc13-Rad5 to form non-canonical K63-linked Ub chains, which leads to an error-free mode of PRR [3]. An *mms2* null mutation causes moderate sensitivity to killing by numerous DNA-damaging agents, a strong synergistic interaction with *rev3*, and a *REV3*-dependent increase in spontaneous mutagenesis [5], [6]. Similar phenotypes have been observed for the *ubc13* null mutant as well [7], [8]. It has long been proposed that error-free PRR utilizes some form of HR to bypass replication-blocking lesions [9]; however, direct evidence only emerged recently for the involvement of HR in error-free PRR [10]. In this report, genes required for HR, including *RAD51*, *RAD52*, *RAD54*, *RAD55* and *RAD57*, were placed downstream of *MMS2* and *UBC13* within the error-free branch of PRR. However, other genes involved in HR, including *MRE11*, *RAD50* and *XRS2*, whose products form a stable complex known as the MRX complex [11], have not been characterized with respect to PRR.

The MRX complex, a member of the structural maintenance of chromosomes (SMC) family of proteins, binds DNA and is known to be involved in numerous activities such as telomere maintenance, DSB recognition and processing, non-homologous end joining, cell cycle checkpoint activation, meiosis and base excision repair [12]–[19]. Mre11 is also known to function as both a single-stranded DNA endonuclease and a 3′-5′ exonuclease [20], [21]. Phenotypically, the null mutant of any one of the MRX components exhibits extreme sensitivity to ionizing radiation and other DNA-damaging agents [11]. Rad50 contains two heptad repeats in its center that fold into a coiled coil [22]. Mre11 binds to the base of the coiled coil (Mre11-Rad50), while at the very tip a conserved Cys-X-X-Cys motif is found to form a hook-shaped domain allowing dimerization with another Mre11-Rad50 dimer resulting in an Mre11_2_Rad50_2_ heterotetramer [23], [24]. Xrs2, the third component of MRX, binds to Mre11 via its conserved C-terminal domain; the interaction between Mre11 and Xrs2 is essential for all known Mre11 functions [25].

Here we report a novel function for the MRX complex in both TLS and error-free PRR. Two relevant nucleases, Exo1 and Sae2, were also characterized in this study. These studies unexpectedly revealed the involvement of the MRX complex in regulating PRR pathways.

## Results

### The MRX complex functions in both TLS and error-free PRR

**Figure 1:**
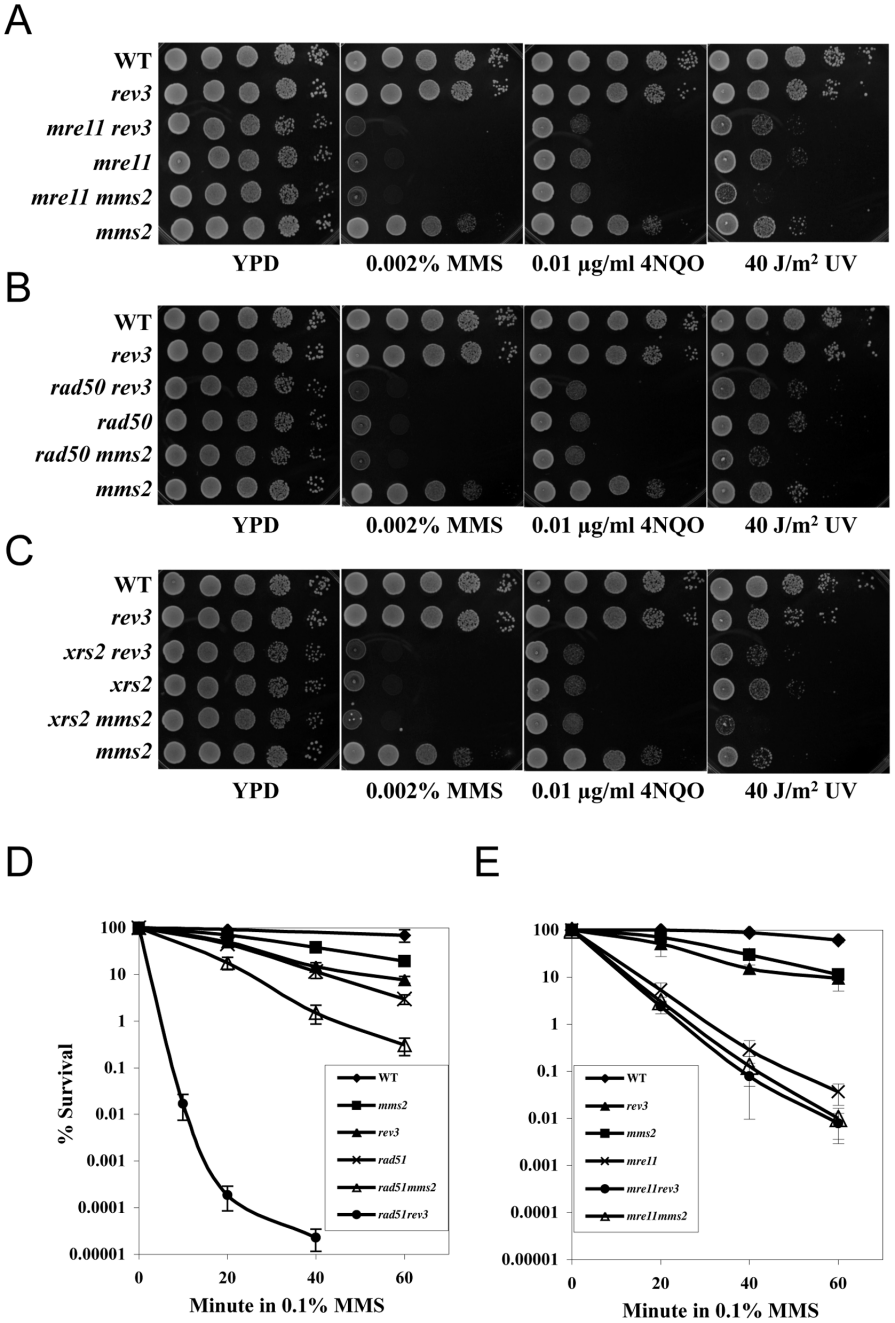
Genetic interactions between *REV3* or *MMS2* and the *MRX* genes with respect to MMS sensitivity. (A–C) Cell survival in a serial dilution assay. Overnight cell cultures were spotted on YPD or YPD containing DNA-damaging agents at the indicated concentration. The plates were incubated at 30°C for 2 days before being photographed. For UV treatment, the YPD plate was exposed to the indicated UV dose and incubated in the dark. All strains used are isogenic to BY4741. It should be noted that for each DNA-damaging agent, several concentrations/doses were examined and only one of the most appropriate concentration/dose is presented for each agent. (A) *mre11 vs. rev3* or *mms2*; (B) *rad50 vs. rev3* or *mms2*; (C) *xrs2 vs. rev3* or *mms2*. (D,E) Cell survival in a liquid killing assay. These results are the average of three independent experiments with standard deviations indicated by error bars. (D) *rad51 vs. rev3* or *mms2*; (E) *mre11 vs. rev3* or *mms2*. All strains used are isogenic to BY4741.

Previous work in our laboratory utilized a synthetic genetic array (SGA) screen [26] of all non-essential genes in *S. cerevisiae* to identify novel genes involved in TLS and error-free PRR [10]. Both *rev1* and *rev3* query strains identified HR genes including *RAD51*, *RAD52*, *RAD54*, *RAD55*, and *RAD57* [10]. Mutations of all the above genes conferred characteristic synergistic interactions with *tls* mutations, while neither the *mms2* nor *ubc13* mutation displayed synergistic interaction with the above HR mutations ([10] and data not shown). To our surprise, none of the *MRX* genes were pulled out in the above SGA screens, suggesting that *mrx* mutations may have unexpected genetic interactions with *tls* mutations. Upon further screening and characterization of the MRX complex, we found that null mutations of *mre11* (Figure 1A), *rad50* (Figure 1B) and *xrs2* (Figure 1C) are essentially epistatic to *rev3* with respect to killing by the alkylating agent methyl methanesulfonate (MMS) that specifically causes replication-blocking lesions, which was in sharp contrast to the synergistic interactions between *hr* and *rev3* mutations [10]. On the other hand, genetic interactions between *mrx* and *mms2* (Figure 1A–C) are comparable to those between *hr* and *mms2* [10]. To further illustrate the differences between *mrx* and *hr* with respect to their genetic interactions with TLS mutations, we performed quantitative liquid killing experiments to compare *rad51* and *mre11*. While *rad51* is indeed synergistic with *rev3* (Figure 1D), the *mre11 rev3* double mutant is barely more sensitive to 0.1% MMS than the *mre11* single mutant (Figure 1E). In addition, while the *mms2 rad51* double mutant is more sensitive to MMS-induced killing than either of the corresponding single mutants (Figure 1D), the *mms2 mre11* double mutant is again barely more sensitive to 0.1% MMS than the *mre11* single mutant (Figure 1E). Similar results were also obtained in response to two other representative DNA-damaging agents, 4-nitroquinoline oxide (4NQO) and UV irradiation (Figure 1A–C). Together these observations suggest that the MRX complex does not function exclusively in error-free PRR like other known HR proteins, and instead functions in both TLS and error-free PRR pathways.

### Genetic interactions between MRX and PCNA modifications

**Figure 2:**
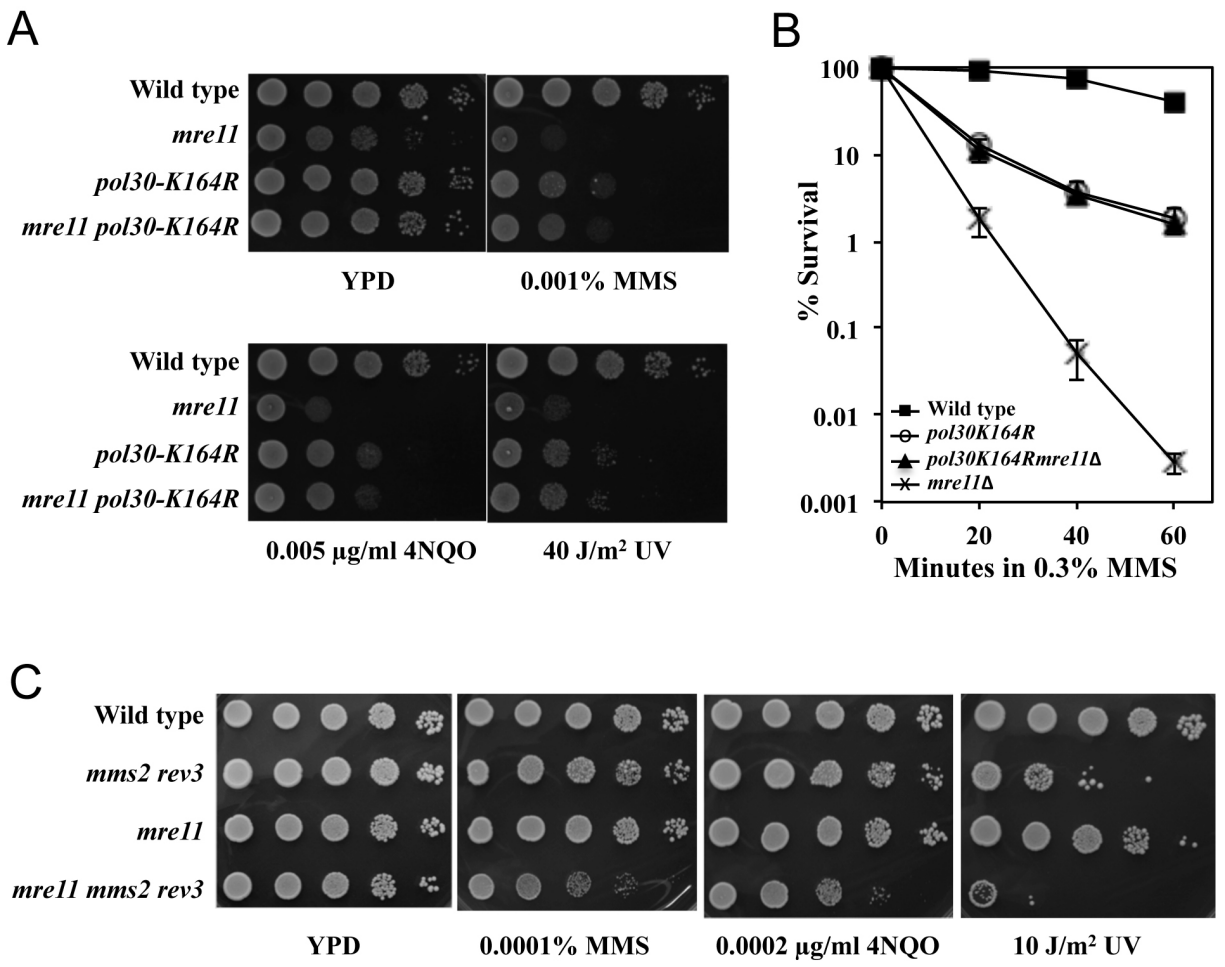
Genetic interactions between *mre11* and PRR pathway mutations. (**A,B**) *pol30-164R* is epistatic to *mre11*. (A) A serial dilution assay as described in Figure 1A. (B) A liquid killing assay. The results are the average of four independent experiments with standard deviations as shown. Yeast strains used: DBY747 (wild type), WXY2379 (*mre11*Δ), WXY2384 (*pol30-K164R*) and WXY2389 (*pol30-K164R mre11*Δ). All strains used are isogenic to DBY747. (C) Genetic interactions between *mre11* and *mms2 rev3* by a serial dilution assay. Experimental conditions were as described in Figure 1A. Yeast strains used: BY4741 (wild type), BY4741 *mre11*Δ, WXY2536 (*rev3*Δ *mms2*Δ) and WXY2528 (*mre11*Δ *rev3*Δ *mms2*Δ). All strains used are isogenic to BY4741.

It is the sequential ubiquitination of PCNA that satisfactorily explains the current genetic observations with regard to how the *RAD6* pathway operates to tolerate and bypass replication-blocking lesions. To critically determine whether MRX genes are involved in the PRR pathways, we combined the *mre11* null mutation with a genomically-integrated *pol30-K164R* point mutation that abolishes PCNA ubiquitination [3]. Our prediction was that if the increased sensitivity conferred by *mre11* were exclusively due to its involvement in PRR, the *mre11 pol30-K164R* double mutant would be as sensitive as one of the single mutants. Indeed, while the *mre11* mutant is more sensitive to MMS than the *pol30-K164R* point mutation, the *mre11 pol30-K164R* double mutant is less sensitive than the *mre11* single mutant and more like the *pol30-K164R* single mutant (Figure 2A). In a liquid killing experiment, the *mre11* null mutant is much more sensitive to MMS than the *pol30-K164R* mutant, but the *mre11* severe sensitivity is completely suppressed by the *pol30-K164R* mutation (Figure 2B). These observations are consistent with the notion that Mre11 functions in the PCNA-K164 ubiquitination-mediated PRR pathway. However, since the PCNA-K164 residue can also be sumoylated [3], which leads to the recruitment of Srs2 helicase and inhibition of HR [27], [28], we cannot rule out the possibility that MRX is also involved in this pathway. Indeed, the *mre11 mms2 rev3* triple mutant is more sensitive to DNA damage than either *mre11* single or the *mms2 rev3* double mutant (Figure 2C), indicating that Mre11 does confer an additional function independent of PCNA mono- and polyubiquitination at the K164 residue.

### The nuclease activity of Mre11 appears to be required for its function in TLS

**Figure 3:**
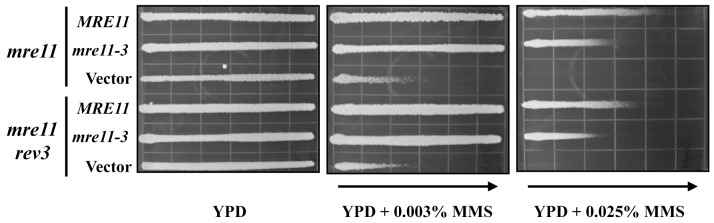
The Mre11 nuclease activity is required for TLS. Single and double mutants were transformed with plasmids carrying wild type, the nuclease/helicase-dead mutations or the vector alone. Overnight cell cultures were imprinted on YPD or YPD+MMS gradient plates at desired concentrations and incubated at 30°C for 2 days before being photographed. Yeast strains used: DBY747 (wild type), WXY2379 (*mre11*Δ) and WXY2390 (*mre11*Δ *rev3*Δ). All strains are isogenic to DBY747.

The MRX complex is well known for its structural function in maintaining sister chromatid cohesion during DNA metabolic events [29]. However Mre11 also maintains a nuclease activity responsible for processing DSB ends and hairpins [20], [30]–[33]. The nuclease activity of Mre11 is not essential for some of its known functions including DNA damage sensitivity [30] and the stabilization of the replisome [34]. In order to determine whether the nuclease activity of Mre11 is required for its function in PRR, we compared the relative sensitivity of a nuclease-deficient *mre11-3* (125–126^HD→LV^) mutant with the *mre11-3 rev3* double mutant. It should be noted that this nuclease-dead mutant is still proficient in allowing the MRX complex to assemble [35] and is much less sensitive to MMS than the *mre11* null mutant (Figure 3). We argue that if the nuclease activity of Mre11 were not required for its function in TLS one would expect to see a synergistic interaction between *mre11-3* and *rev3*. In contrast, the *mre11-3 rev3* double mutant is nearly as sensitive to MMS as the *mre11-3* single mutant (Figure 3), suggesting that the nuclease activity of Mre11 is indeed required for its function in TLS.

### Sae2 is also required for efficient PRR

**Figure 4:**
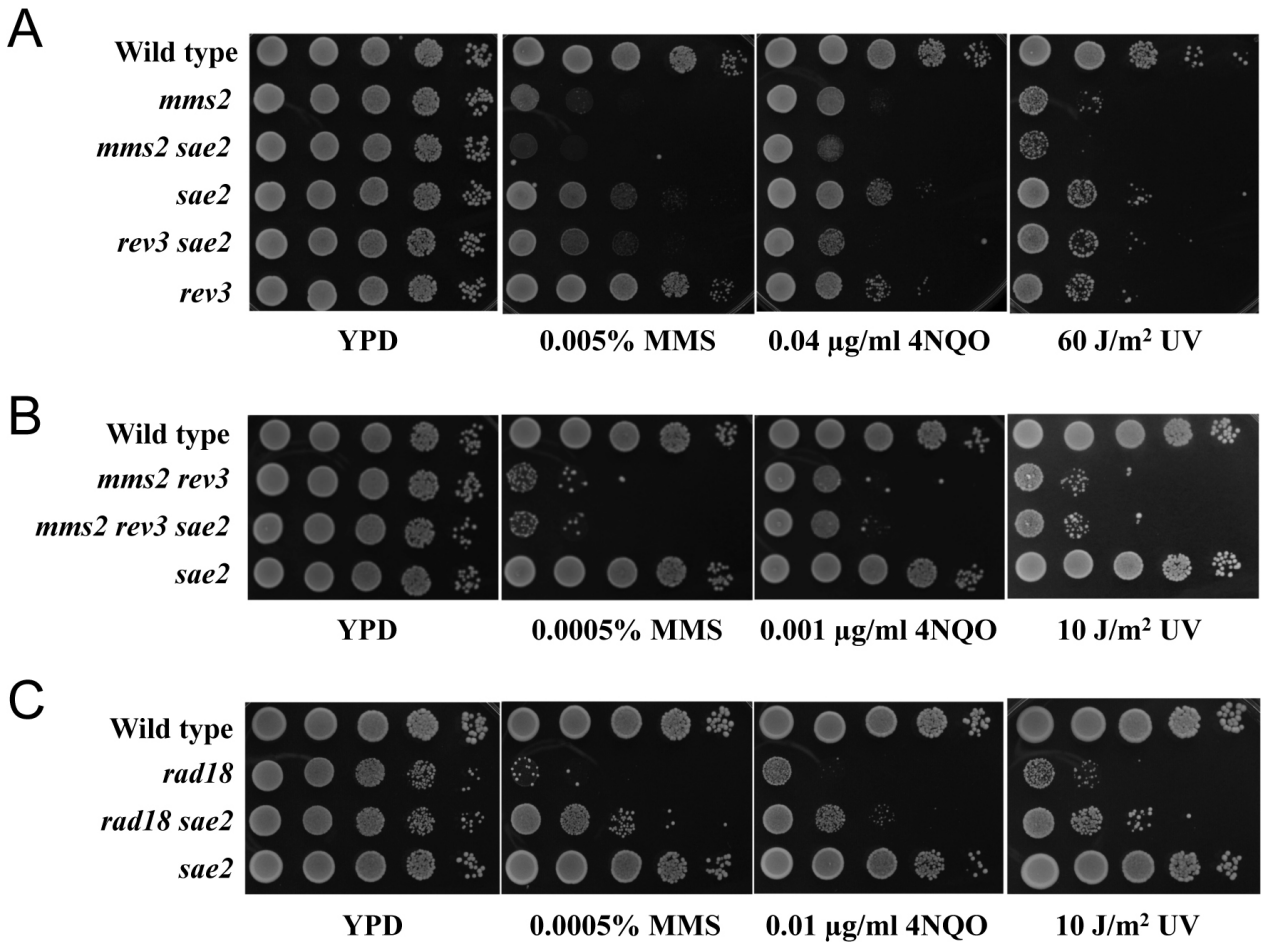
*SAE2* belongs to the yeast PRR pathway. (A,B) *mms2* and *rev3* are epistatic to *sae2* as judged by a serial dilution assay. (A) *sae2* vs. *mms2* or *rev3*. (B) *sae2* vs. *mms2 rev3*. Strains used in (A) and (B) are isogenic derivatives of BY4741. (C) Inactivation of *SAE2* partially rescues *rad18* sensitivity to DNA damage. Strains used in (C) are HK578-10A (wild type) and its isogenic derivatives WXY2975 (*sae2*Δ), WXY930 (*rad18*Δ) and WXY3008 (*rad18*Δ *sae2*Δ). Experimental conditions were as described in Figure 1.

The MRX complex is rapidly recruited to DSBs, signals checkpoint activation and regulates 5′-3′ resection of the DNA ends [15], [36], [37]. MRX is also known to interact with Sae2/CtIP/Ctp1 [38]–[40]. Sae2 was initially discovered in two genetic screens designed to isolate mutants defective in the steps following the initiation of Spo11-induced DSBs but functioning before resolution of the recombination intermediates [41], [42]. Since then Sae2 has been deemed the “unofficial fourth member” of the MRX complex [43]. Similar to the results shown in Figure 1 with *mrx* mutants, the genetic interaction between *sae2* and both *mms2* and *rev3* resulted in double mutations that were either slightly more sensitive than (MMS and 4NQO) or as sensitive as (UV) their respective single mutants (Figure 4A), making it difficult to specifically place *SAE2* in one of the two PRR pathways. To determine whether *SAE2* plays a role in PRR, we deleted *SAE2* in the *mms2 rev3* double mutant and found that the resulting triple mutant was as sensitive to MMS as the *mms2 rev3* double mutant (Figure 4B), suggesting that *SAE2* plays partial roles in both TLS and error-free PRR. To further address whether the increased MMS sensitivity of the *sae2* mutant is due to its role within the PRR pathway, we combined *sae2* with *rad18*, and the double mutants were even less sensitive to MMS, 4NQO or UV than the *rad18* single mutants (Figure 4C). These observations would place *SAE2* within the yeast PRR pathway, although we cannot rule out the remote possibility that genetic relationship between *SAE2* and *RAD18* is due to function(s) of Rad18 independent of PCNA monoubiquitination.

Sae2 controls the initiation of DNA end resection in meiotic and mitotic cells and was recently shown to be a DNA endonuclease [44], a function that is abolished by the *sae2-G270D* mutation. Furthermore, it has been reported that the *sae2-S267A* point mutation, which prohibits the Cdc28-dependent phosphorylation of Sae2, displays a phenotype indistinguishable from the *sae2* null mutant [45]. We found that compared to the *sae2Δ* mutant, *sae2-G270D* and *sae2-S267A* mutants displayed intermediate sensitivity to MMS; when combined with *rev3*, the double mutants were slightly more sensitive to MMS than the *rev3* single mutant (Figure S1 in File S1), suggesting that these activities are also required for the PRR function.

### Exo1 functions in error-free PRR

The Exo1 exonuclease has been implicated in mismatch repair, telomere integrity [46], [47], error-free PRR [48], and more recently long-range resection of DSBs together with MRX and Sae2 [49]–[51]. Therefore, it is necessary to investigate the role of Exo1 in relation to PRR.

**Figure 5:**
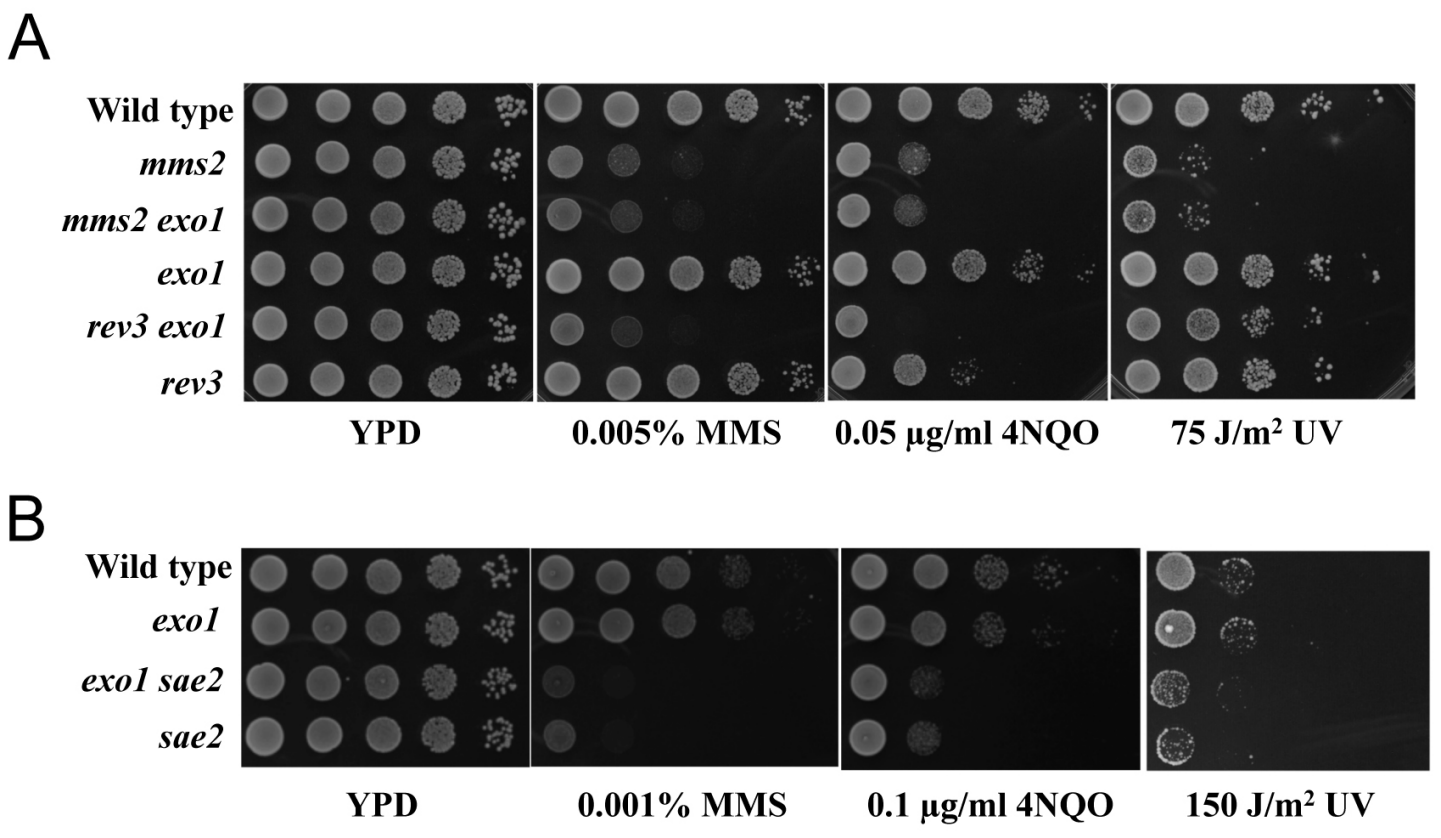
*EXO1* belongs to the error-free PRR pathway. (A) *mms2* is epistatic to *exo1* but *rev3* and *exo1* are additive. (B) *sae2* is epistatic to *exo1*. Strains used are BY4741 and its isogenic derivatives. Experimental conditions were as described in Figure 1.

The *exo1* single mutant does not display noticeable sensitivity to MMS-induced killing (Figure 5), making it difficult to determine its epistatic relationship with known PRR genes. However, the *exo1 rev3* double mutant displays a much greater sensitivity to MMS or 4NQO than either corresponding single mutant (Figure 5A), suggesting that *EXO1* functions in a pathway distinct from TLS. In sharp contrast, the *exo1 mms2* double mutant is as sensitive to MMS as the *mms2* single mutant (Figure 5A), indicating that *EXO1* functions in the error-free PRR pathway, which agrees with a previous report [48]. We also examined the genetic interaction between *SAE2* and *EXO1* and found that the *exo1 sae2* double mutant is as sensitive to MMS as the *sae2* single mutant (Figure 5B). Given the fact that the *exo1* mutation could enhance *rev3* sensitivity, this observation indicates that *sae2* is epistatic to *exo1*, or that, like *EXO1*, *SAE2* also functions in the error-free PRR pathway.

### Spontaneous mutagenesis assays indicate differential involvement of MRX, Sae2 and Exo1 in PRR

Mutations in error-free PRR are characterized by an enhanced spontaneous mutagenesis [5]. If *EXO1* were a member of error-free PRR, its inactivation would be expected to cause an increased spontaneous mutagenesis due to the utilization of TLS. Indeed, deletion of *EXO1* resulted in a 16-fold increase in spontaneous mutagenesis (Table 1). Two observations rule out the possibility that this increase was due to the loss of the mismatch repair activity of *EXO1*. Firstly, the increased mutagenesis seen in the *exo1* mutant was completely dependent on *REV3*, since the *exo1 rev3* double mutant has a spontaneous mutation rate comparable to that of wild-type cells. Secondly, the spontaneous mutation rate in the *exo1 mms2* double mutant is comparable to that of the *mms2* single mutant, which is consistent with a predicted outcome if the enhanced mutagenesis by *exo1* and *mms2* were due to the same mechanisms. Unlike *exo1*, deletion of *MRE11* or *SAE2* did not alter the spontaneous mutation rate over wild-type cells (Table 1), consistent with a notion that they are also required for TLS. This is in sharp contrast to *rad51*, which inactivates HR downstream of error-free PRR [10] and results in a 30-fold increase in spontaneous mutagenesis over wild-type cells.

**Table 1:** Spontaneous mutation rates of *S. cerevisiae* mutants.

Strain^a^	Key alleles	Rate (×10^−8^)^b^	Multiple of wild-type^c^
DBY747	Wild type	0.14±0.12	1
WXY667	*rev3*Δ	0.031±0.014	0.2
WXY2917	*exo1*	2.27±0.63	16.2
WXY644	*mms2*Δ	2.72±0.64	19.4
WXY2394	*sae2*Δ	0.18±0.08	1.3
WXY2397	*mre11*Δ	0.16±0.07	1.1
WXY1164	*rad51*Δ	4.2±0.6	30.0
WXY2918	*exo1*Δ *mms2*Δ	3.33±0.3	23.8
WXY2991	*exo1*Δ *rev3*Δ	0.12±0.07	0.9

a All strains are isogenic derivatives of DBY747.

b The spontaneous mutation rates are the average of at least three independent experiments with standard deviation.

c Rate relative to the wild-type mutation rate.

### Effects of *mre11, sae2* and *exo1* on PCNA ubiquitination

The epistatic relationship between *mre11* and *pol30-K164R* as shown in Figure 2 does not necessarily indicate whether the MRX complex acts upstream or downstream of PCNA ubiquitination. To answer this question, we set out to determine if deletion of *MRX* genes alters the relative level of PCNA ubiquitination. A series of experiments as shown in Figures S2 and S3 in File S1 confirm that we were able to detect mono-and di-ubiquitinated PCNA in the yeast whole cell extract without the need for a prior affinity purification.

**Figure 6:**
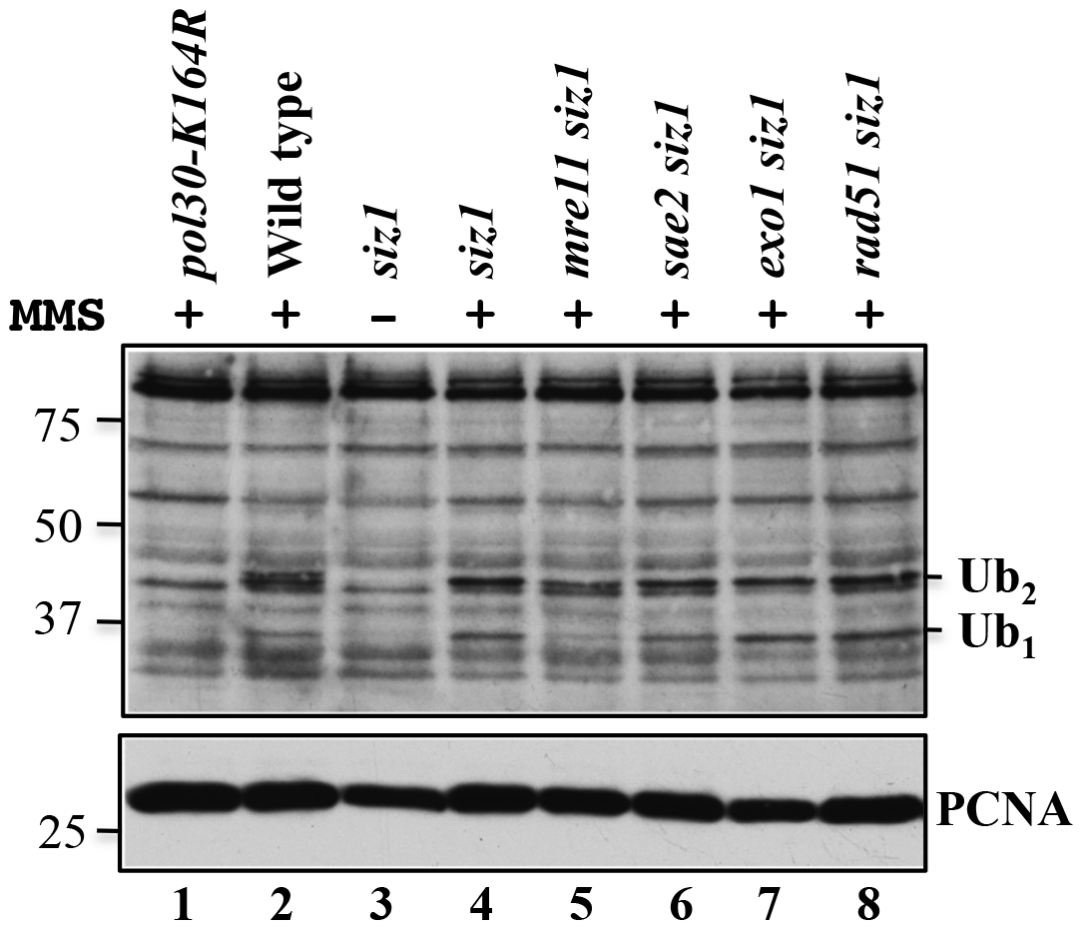
Effects of *mre11*, *sae2*, *exo1* and *rad51* on MMS-induced mono- and diubiquitination of PCNA. Overnight cultures were subcultured and allowed to grow to a cell count of approximately 1×10^7^cells/ml before being treated with 0.05% MMS (as indicated) for 90 minutes. Total cell extracts were obtained under denaturing conditions and analyzed by SDS-PAGE and western blot. Strains used were HK578-10A (wild type) and its isogenic derivatives WXY994 (*pol30-K164R*), WXY2959 (*siz1*Δ), WXY2995 (*mre11*Δ *siz1*Δ), WXY2962 (*sae2*Δ *siz1*Δ), WXY2963 (*exo1*Δ *siz1*Δ) and WXY2994 (*rad51*Δ *siz1*Δ). Ub_1_ refers to monoubiquitinated PCNA. Ub_2_ refers to diubiquitinated PCNA.

We repeatedly observed a drastic decrease in monoubiquitinated PCNA in an *mre11 siz1* mutant compared to the *siz1* and *rad51* mutants (Figure 6, cf. lanes 4, 5 and 8). *rad51* is not expected to alter PCNA ubiquitination as it has only been suggested to function downstream of error-free PRR [10]. In contrast, deletion of *MRE11* almost completely abolishes MMS-induced PCNA monoubiquitination (cf. lanes 4 and 5) and meanwhile reduces the level of diubiquitinated PCNA by almost 1/3 (cf. lanes 4 and 5), suggesting that the MRX complex is a novel member of the PRR pathway functioning upstream of PCNA ubiquitination.

Genetic analysis does not clearly assign Sae2 to the error-free or TLS PRR pathway; indeed deletion of *SAE2* does not appear to significantly alter the levels of mono- or diubiquitinated PCNA. Deletion of *exo1* decreases the level of diubiquitinated PCNA by approximately 35% with a corresponding increase in monoubiquitinated PCNA (Figure 6, lane 7), lending further support to the notion that Exo1 plays an accessory role in error-free PRR. Collectively, the above observations allow us to conclude that MRX, Sae2 and Exo1 are variably required for PCNA ubiquitination while some of them play multiple roles in PRR.

### Physical interaction between Rad18 and the MRX complex

**Figure 7:**
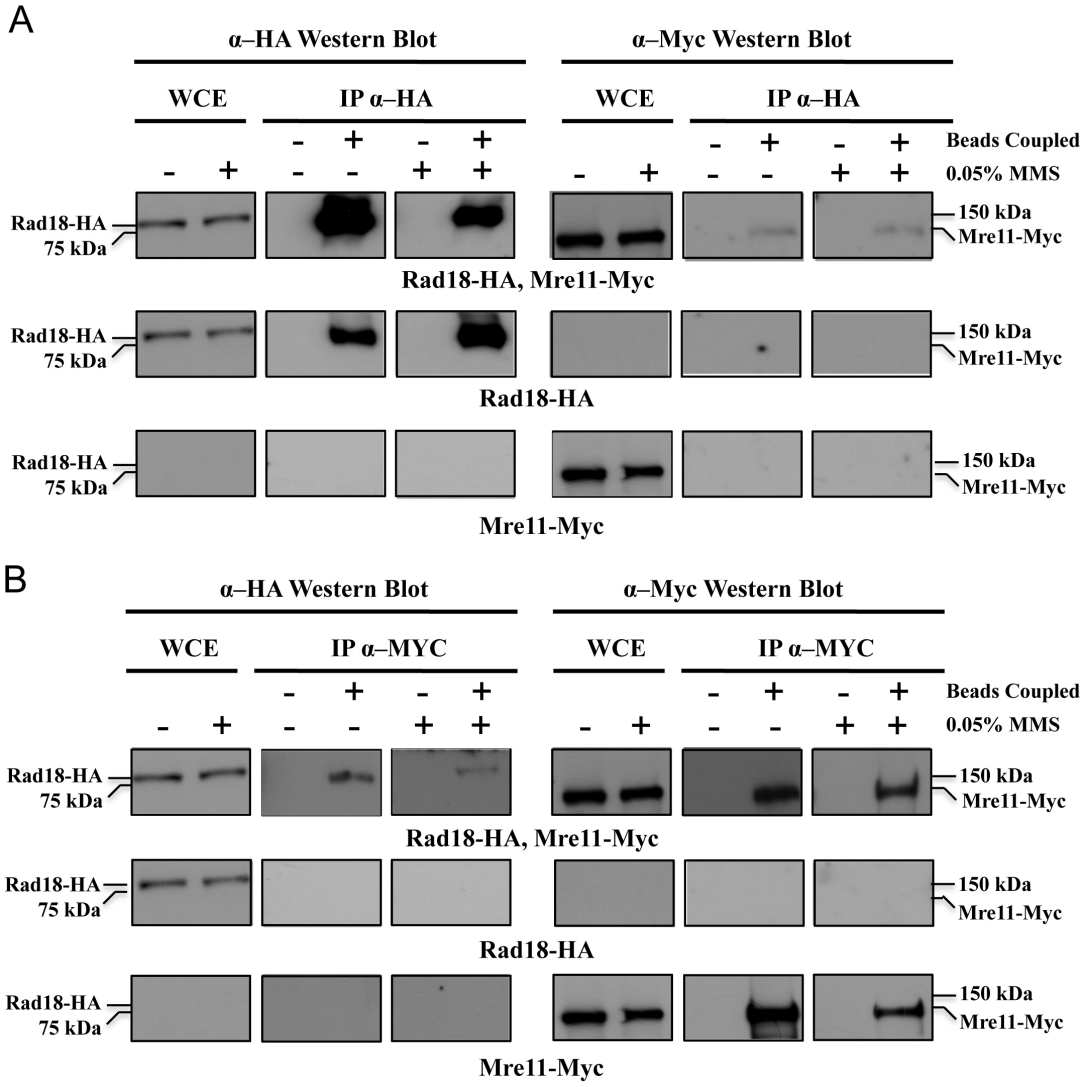
Mre11 physically interacts with Rad18 *in vivo*. Asynchronous W303 tagged yeast strains containing either Rad18-HA and Mre11-Myc, Rad18-HA alone, or Mre11-Myc alone were used for analysis in this experiment. Strains containing only Rad18-HA or only Mre11-Myc tags were used as negative controls. Cells were grown to 1×10^7^ cells/ml before being treated with or without 0.05% MMS (as indicated) for 90 minutes. Cells were then cross-linked with formaldehyde prior to cell lysis and the lysates were immunoprecipitated with either (A) anti-HA antibody, or (B) anti-Myc antibody. Lysates were also incubated with uncoupled beads as another negative control as shown in A and B. Whole cell extracts and immunoprecipitates were then analyzed by western blot analysis with anti-Myc and anti-HA antibodies.

Our observation that inactivation of *MRE11* drastically reduces PCNA monoubiquitination suggests that the MRX complex modulates the Rad6-Rad18 activity required for PCNA monoubiquitination. To look into mechanistic insights of this regulation, we asked if the MRX complex physically interacts with Rad6-Rad18 by a cross-linked co-immunoprecipitation (co-IP) assay essentially as previously described [52]. First, HA-tagged Rad18 was precipitated with an anti-HA antibody from cells with or without 0.05% MMS treatment for 90 minutes. Myc-tagged Mre11 was then examined from the co-precipitates by western blot analysis. Our results reproducibly demonstrated an interaction between Rad18 and Mre11 both in the presence and absence of DNA damage (Figure 7A). The same specific interaction was also observed in the reverse co-IP experiment (Figure 7B). Hence, the MRX complex may be constitutively associated with Rad6-Rad18. We noted a decrease in immunoprecipitated Rad18-HA after MMS treatment, regardless of being used as a bait or prey. Since the total amount of Rad18-HA remains the same before and after MMS treatment, we suspect that it is due to MMS-induced S-phase cell cycle arrest that alters Rad18-HA immunoprecipitation, possibly through a conformational change.

## Discussion

Here we report that MRX, Sae2 and Exo1 endo/exonucleases are variably involved in the error-prone and error-free branches of PRR. This study offers a greater understanding of how TLS and error-free PRR are co-ordinately operated at the molecular level.

MRX has been implicated in numerous DNA damage response pathways specifically in the processing of DSBs during meiosis and mitosis. It would be highly expected for MRX to play a role downstream of error-free PRR along with other HR proteins [10]. However, in addition to its expected genetic interactions with members of error-free PRR, *mrx* mutations are surprisingly epistatic to mutations in the TLS pathway. The involvement of MRX in TLS was further confirmed by several observations. First of all, unlike other *HR* genes, none of the *MRX* genes were identified from a conditional synthetic lethal screen using either TLS or error-free PRR pathway mutants as queries; the absence of synergistic interactions was later individually confirmed. Secondly, the *pol30-K164R* mutation is epistatic to *mre11*, indicating that the DNA damage tolerance to MMS conferred by the MRX complex is completely dependent on PCNA covalent modifications at the K164 residue. Thirdly, despite numerous roles played by MRX to maintain genomic stability, deletion of *MRE11* does not result in an increased spontaneous mutagenesis in a *trp1-289* reversion assay, which is tailored to detect base substitutions. This is in sharp contrast to *hr* mutants like *rad51*. Fourthly, deletion of *MRE11* noticeably reduces levels of both mono- and diubiquitination of PCNA. Finally, we have shown that Rad18 binds to Mre11 *in vivo*, providing direct physical evidence that the MRX complex is a novel member of the PRR pathway and is required for both branches of PRR. It is of great interest to note a report that in mammalian cells, NBS1, the yeast Xrs2 homolog, interacts with RAD18 following UV irradiation, recruiting RAD18 to sites of DNA damage [53].

Sae2 is considered an accessory factor of the MRX complex during DSB resection. Although *sae2* does not display a clear epistasis relationship with either *mms2* or *rev3*, we argue that this observation is a result of Sae2 being partially required for both PRR pathways. This argument is further supported by several observations. Firstly, although *sae2* is slightly additive to *mms2* or *rev3* individually, when both *MMS2* and *REV3* are inactivated in a double mutant further deletion of *SAE2* does not cause increased sensitivity to MMS. Secondly, both *rad18* and *pol30-K164R* are epistatic to *sae2*, indicating that once PCNA cannot be ubiquitinated, *SAE2* plays no role in the protection of host cells from MMS-induced DNA damage. Thirdly, like *mre11*, the *sae2* mutant does not display increased spontaneous mutagenesis, consistent with a role in TLS. Fourthly, *sae2* is epistatic to *exo1*, suggesting that Sae2 must play an overlapping role with Exo1 within error-free PRR. Finally, careful examination of PCNA ubiquitination indicates that deletion of *SAE2* partially reduces both mono- and diubiquitinated PCNA, albeit to a lesser extent than *mre11*. These observations are consistent with Sae2 being an accessory protein for MRX within PRR pathways.

The Exo1 exonuclease is also a multi-functional protein and its involvement in error-free PRR was reported by means of epistasis analyses [48]. Supporting this conclusion is the observation that *exo1* and *rad9* are synergistic [48], a characteristic trait of an error-free PRR component [54]. Consistent with this, we find that deletion of *EXO1* results in a dramatic increase in spontaneous mutations in a *trp1-289*-based mutagenesis assay and this increase is largely dependent on functional *REV3* and due to defective error-free PRR. Remarkably, deletion of *EXO1* specifically compromises the relative level of diubiquitinated PCNA without affecting its monoubiquitination. Hence, Exo1 is exclusively involved in the error-free PRR branch. Given the fact that the *exo1* single mutant barely displays an increased sensitivity to MMS, we suspect that Exo1 only plays an accessory role in the promotion of error-free PRR.

**Figure 8:**
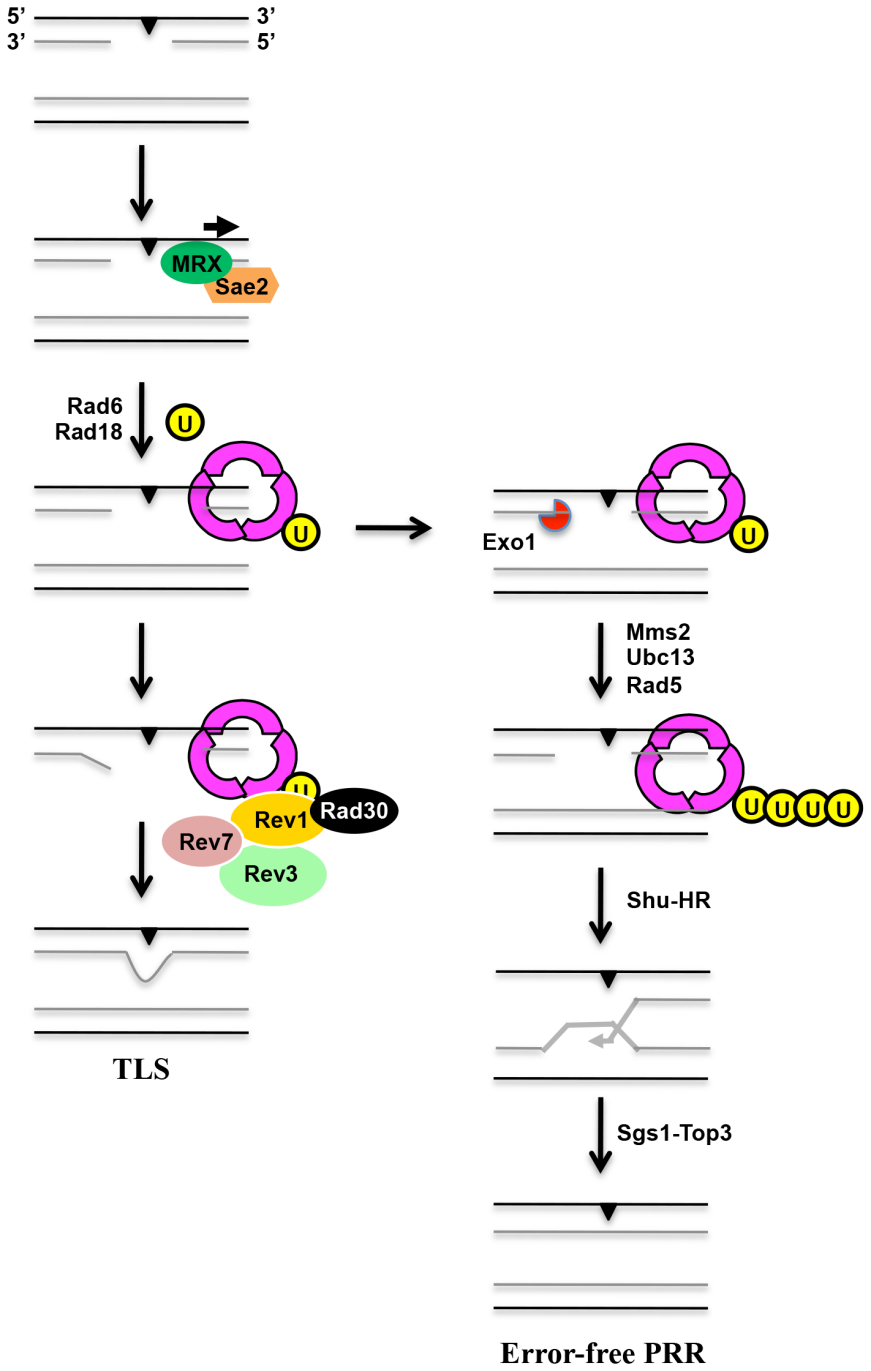
A proposed working model for the budding yeast PRR pathways. MRX, in conjunction with Sae2, functions upstream of PCNA monoubiquitination by ssDNA resection thus promoting Rad6-Rad18 to monoubiquitinate PCNA. Exo1 processes ssDNA gaps in the 5′-3′ direction, which facilitates PCNA polyubiquitination by Rad5-Ubc13-Mms2 and subsequent error-free lesion bypass mediated by the Shu complex, HR and Sgs1-Top3 resolution.

The involvement of MRX, Sae2 and Exo1 in the different modes of PRR is highly surprising and unexpected. When this research was in progress, several laboratories independently reported differential involvement of the above proteins in the sequential processing of DSB ends [49]–[51], which shed light on the possible co-ordination of these proteins in the PRR pathway. We argue that to apply the DSB processing model to PRR, one has to first ask whether the nuclease activities of the above proteins are required for PRR. Collectively our results suggest that these enzymatic activities are critical for PRR. Secondly, we envisage that the major difference between the DSB model and PRR is that the latter acts on ssDNA gaps. This may not pose a problem since based on the DSB processing model, the above enzymes primarily act at the junction of single-double stranded DNA. With the above possibility in mind, it is of great interest to note a recent report [55] in which yeast and frog Rad51 is shown to protect Mre11-dependent nascent DNA degradation at or behind replication forks. Thirdly, the long-range DSB end processing model only deals with 5′-3′ resection, whereas it is unclear whether this is the only orientation of processing for PRR. Nevertheless, it is noticed that the Mre11 subunit of MRX possesses a 3′-5′ exonuclease activity [20], which has not been fully accounted for by the DSB processing model. By our genetic and physical analyses and inference to the DSB processing model, we propose that MRX and Sae2 participate in the initial processing of ssDNA gaps, and the recruitment of PRR proteins by binding to Rad18, all of which is required for efficient PCNA ubiquitination and lesion bypass. In contrast, Exo1 only promotes error-free PRR, perhaps by signalling for polyubiquitination. A working model of PRR based on previous reports and the above analyses is presented in Figure 8. According to this model, the MRX complex functions upstream of PCNA to resect ssDNA at the stalled replication fork. Sae2 may facilitate MRX activity by removing DNA-binding proteins [56] or secondary structures [44]. The binding of the MRX complex to Rad18 recruits Rad6-Rad18 [57], which monoubiquitinates PCNA for efficient lesion bypass via TLS. On the other hand, the 5′-3′ exonuclease activity of Exo1 causes further strand resection that favours the recruitment of Rad5-Ubc13-Mms2 to polyubiquitinate PCNA and allows for error-free PRR lesion bypass via the Shu complex, HR and Sgs1-Top3. As all the genes described in this report are conserved in eukaryotes, from yeast to human, it would be of great interest to determine if the same regulatory mechanisms occur in higher eukaryotes.

## Materials and Methods

### Yeast strains and culture

The *S. cerevisiae* yeast strains used in this study are listed in Table S1 in File S1. All of the strains are isogenic derivatives of DBY747, HK578 or BY4741. HK578 is a derivative of W303 and has been corrected for the *RAD5* gene by Dr. H. Klein (New York University). The ORF deletion strains of BY4741 were created by the *Saccharomyces* Genome Deletion Project Consortium and purchased from Research Genetics (Invitrogen, Carlsbad, CA, USA).

Yeast cells used in this study were cultured at 30°C in either rich YPD medium, or an SD medium supplemented with essential nutrients as required [58] unless otherwise specified. Yeast cells were transformed via a modified lithium acetate method [59]. Yeast strains were created as a result of synthetic genetic array (SGA) crosses, or by a one-step targeted gene deletion using a disruption cassette. Newly created strains were confirmed via phenotypic change when possible, and by PCR of genomic DNA. Sources and use of disruption cassettes *rad51*Δ::*LEU2* [10], *mms2*Δ*::LEU2* [5], *rev3*Δ::*LEU2* [60], *rev3*Δ::*hisG-URA3-hisG* [61] and *mre11*Δ::*HIS3* [62] have been previously described. For *EXO1* disruption, the 2.1-kb *EXO1* ORF was cloned into pBluescript and the 1.3-kb *Nde*I-*Bsa*BI fragment within the *EXO1* ORF was deleted and replaced by a *Bam*HI linker, which was then used to clone either a 1.6-kb *Bam*HI fragment containing *LEU2* from YDp-L or a 1.1-kb *Bam*HI fragment containing *URA3* from YDp-U [63]. The *exo1*Δ::*LEU2* disruption cassette was released by *Bgl*II-*Pst*I digestion and the *exo1*Δ::*URA3* disruption cassette was released by *Bgl*II-*Sna*BI digestion prior to yeast transformation. For *SAE2* disruption, a 1.7-kb yeast genomic DNA fragment containing the *SAE2* ORF and flanking regions was amplified by primers SAE2-1 (5′-GGG CTG CAG TGT ACT TAG CCG TTC-3′) and SAE2-2 (5′-GCG AAA ATA ACG TCG ACG TTC-3′) and cloned into pGEM-T. A 1.0-kb *Hin*dIII-*Bsi*WI fragment containing essentially the entire *SAE2* ORF was deleted and replaced by a *Bam*HI linker, which was used to clone the 1.6-kb *Bam*HI fragment containing *LEU2* from YDp-L [63] to form p*sae2*Δ::LEU2. The *sae2*Δ::*LEU2* disruption cassette was released by *Pst*I-*Sal*I digestion prior to yeast transformation. For *SIZ1* disruption, a 2.0-kb yeast genomic DNA fragment within the *SIZ1* ORF was amplified by primers SIZ1-3 (5′-CAG AAA GAA TGA ACC TTT GCC-3′) and SIZ1-4 (5′-GTG GAA GGA AAG GAC ATA TCC-3′) and cloned into pGEM-T. A 1.4-kb *Bam*HI fragment was deleted and replaced by either a 1.16-kb *Bam*HI fragment containing *HIS3* from YDp-H or a 1.1-kb *Bam*HI fragment containing *URA3* from YDp-U [63]. The *siz1*Δ::*HIS3* disruption cassette was released by *Apa*LI-*Eco*RV digestion and the *siz1*Δ::*URA3* disruption cassette was released by *Bgl*II-*Cla*I digestion prior to yeast transformation.

### Testing for sensitivity to DNA-damaging agents

Gradient plate assays were used as a semi-quantitative measurement of relative MMS sensitivity as previously described [64]. The MMS-induced liquid killing experiment was conducted as previously described [60]. Briefly, overnight yeast cultures were used to inoculate fresh YPD and grown at 30°C until a cell count of approximately 2×10^7^cells/ml was achieved. MMS was then added to the liquid culture and samples were taken at the indicated times. Cells were pelleted by centrifugation, washed, diluted, and plated on YPD. Colonies were counted after 3 days of incubation and scored as a percentage of cell survival against untreated cells.

### Spontaneous mutagenesis assay

The spontaneous mutation rate was measured by monitoring the Trp^+^ reversions of the *trp1-289* allele in the DBY747 strain via a modified Luria and Delbruck fluctuation test as previously described [64].

### Detection of PCNA ubiquitination

Detection of ubiquitinated PCNA was adapted from a previous report [65]. Briefly, cells grown overnight in YPAD (YPD+20 mg/ml Ade) were diluted to 0.3×10^7^cells/ml in 100 mls of YPAD and allowed to grow for an additional 2 hours. Cultures were then split and one was treated with 0.05% MMS for 90 minutes. Cells were harvested and immediately frozen in liquid nitrogen for 10 minutes. After step-wise N-ethylmaleimide (NEM) treatment plus phenylmethylsulfonyl fluoride (PMSF), NaOH plus 7.5% β-mercaptoethanol incubation and trichloroacetic acid precipitation. The pellet was then resuspended in a modified HU buffer (8 M Urea, 5% SDS, 200 mM Tris-HCL pH 6.8, 1 mM EDTA, 0.025% bromophenol blue, 1.5% DTT, 25 mM NEM, 1 mM PMSF, and 0.5% triton-X-100) prior to the protein heat denaturation. Samples were then added to the Bio-Rad laemmli sample buffer, frozen overnight and analyzed by SDS-PAGE and western blotting. Anti-Pol30 monoclonal antibodies were raised and characterized in-house. Quantitative analysis of mono- and diubiquitinated PCNA was accomplished with Quantity One 4.4.1 software. Mean values were corrected for background, and analyzed as a percentage of the *siz1* null mutation. This percentage was then corrected for loading control and the strain treated with MMS containing the *pol30-K164R* point mutation was corrected to 0%. MMS-treated *siz1* null was treated as 100% for both mono- and diubiquitinated PCNA. Results were then graphed.

### Co-immunoprecipitation

The cross-linked immunoprecipitation assay was performed essentially as described [66]. Cells were grown overnight at 30°C in 100 ml YPAD to 1.0×10^7^/ml and treated with 0.05% MMS for 90 minutes or remained untreated. After cells were treated with 1% formaldehyde for 20 minutes at 30°C with shaking, 2.5 ml of 2.5 M glycine was added for 5 minutes at 30°C with shaking before cells were pelleted and washed twice with 20 ml ice-cold TBS (20 mM Tris-HCl, pH 7.5, 150 mM NaCl). Pellets were then resuspended in 0.8 ml of lysis buffer (50 mM HEPES-KOH, pH 7.5, 140 mM NaCl, 1% Triton-X-100, 0.1% sodium deoxycholate, and 1 complete protease inhibitor pellet), transferred to a 2-ml screw-cap tube, and ∼600 µl of Zirconia/Silica beads were added. Cells were bead-beaten and sonicated to reduce the DNA size, and added to either anti-HA (Sigma F-7)-coupled dynabeads, or uncoupled beads. Immunoprecipitations were allowed to incubate at 4°C for a minimum of 2 hours before the beads were washed with the lysis buffer containing 0.5 M NaCl, followed by two washes with 1 ml of wash buffer (10 mM Tris-HCl, pH 8.0, 250 mM LiCl, 0.5% NP-40, 0.1% sodium deoxycholate, 1 mM EDTA, 1 complete protease inhibitor pellet). After a final wash with 1 ml of lysis buffer, the beads were resuspended in 40 µl elution buffer (50 mM Tris-HCl, pH 8.0, 10 mM EDTA, 1% SDS), and 40 µl of laemmli sample buffer before being frozen at −20°C overnight. Samples were incubated at 99°C for 30 minutes before being run on an 8% SDS-PAGE gel and analyzed by western blotting with anti-HA and anti-MYC (9E10) antibodies.

## Supporting Information

File S1 **Table S1**, *Saccharomyces cerevisiae* strains. **Figure S1**, Gradient plate assay showing that the nuclease activity of Sae2 plays a role in PRR. Single and double mutants were transformed with plasmids carrying wild type, the nuclease/helicase-dead mutations or the vector alone. Overnight cell cultures were imprinted on YPD or YPD+MMS at desired concentrations and incubated at 30°C for 2 days before being photographed. Strains used were isogenic to BY4741. **Figure S2**, Control experimental data to confirm anti-PCNA antibody and detection of PCNA ubiquitination. Overnight cultures were subcultured and allowed to grow to a cell count of approximately 1×10^7^ cells/ml before being treated with 0.05% MMS (as indicated) for 90 minutes. Total cell extracts were obtained under denaturing conditions and analyzed by SDS-PAGE and western blot. (A) Monoubiquitinated PCNA is detected in wild-type yeast whole cell extracts without the need for His_n_-affinity purification. The PCNA ubiquitination band is slightly shifted up in the strain containing the Pol30-His_7_ allele compared to the native Pol30 allele (cf. lanes 5 and 6) further confirms that this band is PCNA modification. (B) Overexpression of Rad6 and/or Rad18 enhances detection of PCNA monoubiquitination; however, it is not required for the detection of monoubiquitination (cf. lanes 5 and 6). (C) A null mutation of *rad18* abolishes monoubiquitinated PCNA. Strains used were HK578-10A (wild-type) and its isogenic derivatives WXY994 (*pol30-K164R*) and WXY930 (*rad18*Δ). **Figure S3**, Control experiments to confirm di-ubiquitination of PCNA. (A) SUMOylated PCNA is observed in the absence of MMS treatment (lanes 1 and 3), but it is dependent on the Pol30-K164 residue (lanes 2 and 4), as well as *SIZ1* (lane 5). (B) Upon MMS treatment, the two prominent bands marked as Ub_1_ and Ub_2_ are deemed to be PCNA mono- and diubiquitinations, respectively, as they were shifted in the lane containing the Pol30-His_7_ cell extract (cf. lanes 1 and 3), and were abolished in the *pol30-K164R* mutations (lanes 2 and 4). As expected, they were not affected by deletion of *SIZ1* (lane 5) and only the diubiquitinated PCNA was abolished by the *mms2* null mutation (lane 6). Strains used were HK578-10A (wild-type) and its isogenic derivatives WXY994 (*pol30-K164R*), WXY2959 (*siz1*Δ) and WXY2960 (*mms2*Δ *siz1*Δ).
(DOCX)
