# Supplementary material for: The Mre11-Rad50-Xrs2 Complex Is Required for Yeast DNA Postreplication Repair
Source: PLoS One. 2014 Oct 24;9(10):e109292. doi: 10.1371/journal.pone.0109292 (PMC4208732; doi:10.1371/journal.pone.0109292)
Supplement: File S1 — Table S1, Saccharomyces cerevisiae strains. Figure S1, Gradient plate assay showing that the nuclease activity of Sae2 plays a role in PRR. Single and double mutants were transformed with plasmids carrying wild type, the nuclease/helicase-dead mutations or the vector alone. Overnight cell cultures were imprinted on YPD or YPD+MMS at desired concentrations and incubated at 30°C for 2 days before being photographed. Strains used were isogenic to BY4741. Figure S2, Control experimental data to confirm anti-PCNA antibody and detection of PCNA ubiquitination. Overnight cultures were subcultured and allowed to grow to a cell count of approximately 1×10^7^ cells/ml before being treated with 0.05% MMS (as indicated) for 90 minutes. Total cell extracts were obtained under denaturing conditions and analyzed by SDS-PAGE and western blot. (A) Monoubiquitinated PCNA is detected in wild-type yeast whole cell extracts without the need for Hisn-affinity purification. The PCNA ubiquitination band is slightly shifted up in the strain containing the Pol30-His7 allele compared to the native Pol30 allele (cf. lanes 5 and 6) further confirms that this band is PCNA modification. (B) Overexpression of Rad6 and/or Rad18 enhances detection of PCNA monoubiquitination; however, it is not required for the detection of monoubiquitination (cf. lanes 5 and 6). (C) A null mutation of rad18 abolishes monoubiquitinated PCNA. Strains used were HK578-10A (wild-type) and its isogenic derivatives WXY994 (pol30-K164R) and WXY930 (rad18Δ). Figure S3, Control experiments to confirm di-ubiquitination of PCNA. (A) SUMOylated PCNA is observed in the absence of MMS treatment (lanes 1 and 3), but it is dependent on the Pol30-K164 residue (lanes 2 and 4), as well as SIZ1 (lane 5). (B) Upon MMS treatment, the two prominent bands marked as Ub1 and Ub2 are deemed to be PCNA mono- and diubiquitinations, respectively, as they were shifted in the lane containing the Pol30-His7 cell extract (cf. lanes 1 and 3), and were a [file pone.0109292.s001.docx]

**Combined Supporting Information File S1**

**Table S1**: *Saccharomyces cerevisiae* strains

| Strain | Genotype | Source |
| --- | --- | --- |
| BY4741 | *MAT***a** *his3*∆1 *leu2*∆0 *met15*∆0 *ura3*∆0 | SGD Consortium |
| BY4741 *trp1*∆ | BY4741 with *trp1*∆::Kan^R^ | SGD Consortium |
| BY4741 *rev3*∆ | BY4741 with *rev3*∆::Kan^R^ | SGD Consortium |
| BY4741 *xrs2*∆ | BY4741 with *xrs2*∆::Kan^R^ | SGD Consortium |
| BY4741 *mre11*∆ | BY4741 with *mre11*∆::Kan^R^ | SGD Consortium |
| BY4741 *mms2*∆ | BY4741 with *mms2*∆::Kan^R^ | SGD Consortium |
| BY4741 *rad50*∆ | BY4741 with *rad50*∆::Kan^R^ | SGD Consortium |
| BY4741 *rad51*∆ | BY4741 with *rad51*∆::Kan^R^ | SGD Consortium |
| BY4741 *rad52*∆ | BY4741 with *rad52*∆::Kan^R^ | SGD Consortium |
| BY4741 *rad54*∆ | BY4741 with *rad54*∆::Kan^R^ | SGD Consortium |
| BY4741 *rad55*∆ | BY4741 with *rad55*∆::Kan^R^ | SGD Consortium |
| BY4741 *rad57*∆ | BY4741 with *rad57*∆::Kan^R^ | SGD Consortium |
| BY4741 *exo1*∆ | BY4741 with *exo1*∆::Kan^R^ | SGD Consortium |
| BY4741 *sae2*∆ | BY4741 with *sae2*∆::Kan^R^ | SGD Consortium |
| WXY2469 | BY4741 with *mms2*∆::Kan^R^ *rad51*∆*LEU2* | This Study |
| WXY2950 | BY4741 with *sae2*∆::Kan^R^ *exo1*∆*LEU2* | This Study |
| WXY2547 | BY4741 with *sae2*∆::Kan^R^ *mms2*∆*LEU2* | This Study |
| WXY2546 | BY4741 with *sae2*∆::Kan^R^ *rev3*∆*LEU2* | This Study |
| WXY2949 | BY4741 with *mms2*∆::Kan^R^ *exo1*∆*LEU2* | This Study |
| WXY2551 | BY4741 with *rev3*∆::Kan^R^ *exo1*∆::*LEU2* | This Study |
| WXY2536 | BY4741 with *rev3*∆::Kan^R^ *mms2∆::URA3* | Lab Stock |
| WXY2528 | BY4741 with *mre11*∆::Kan^R^ *rev∆∆*::nat^R^ *mms2∆::LEU2* | This Study |
| Y5565 | *MAT*α *can1*∆::*MFA1pr-HIS3* *mfa1*∆::*MF*α*1pr-LEU2 lyp1*∆ | Lab Stock |
|  | *his3*∆*1 leu2*∆*0 ura3*∆*0 met15*∆*0* |  |
| Y8621 | Y5565 with *rev3*∆::*nat^R^ lys2*∆+ | Lab Stock |
| Y9897 | Y5565 with *mms2*∆::*nat^R^ lys2*∆+ | Lab Stock |
| DMA (deletion mutant array) | BY4741 *MAT***a** *can1*∆::*MFA1pr-HIS3 lyp1*∆ |  |
| WXY2491 | DMA with *rev3*∆::nat^R^ *mre11*∆an^R^ | This Study |
| WXY2493 | DMA with *mms2*∆::nat^R^ *mre11*∆an^R^ | This Study |
| WXY2460 | DMA with *rev3*∆::nat^R^ *rad50*∆an^R^ | This Study |
| WXY2462 | DMA with *mms2*∆::nat^R^ *rad50*∆an^R^ | This Study |
| WXY2219 | DMA with *rev3*∆::nat^R^ *rad51*∆an^R^ | This Study |
| WXY2416 | DMA with *mms2*∆::nat^R^ *rad52*∆an^R^ | This Study |
| WXY2032 | DMA with *rev3*∆::nat^R^ *rad52*∆an^R^ | This Study |
| WXY2417 | DMA with *mms2*∆::nat^R^ *rad54*∆an^R^ | This Study |
| WXY2555 | DMA with *rev3*∆::nat^R^ *rad54*∆an^R^ | This Study |
| WXY2419 | DMA with *mms2*∆::nat^R^ *rad55*∆an^R^ | This Study |
| WXY2020 | DMA with *rev3*∆::nat^R^ *rad55*∆an^R^ | This Study |
| WXY2420 | DMA with *mms2*∆::nat^R^ *rad57*∆an^R^ | This Study |
| WXY2421 | DMA with *rev3*∆::nat^R^ *rad57*∆an^R^ | This Study |
| WXY2477 | DMA with *rev3*∆::nat^R^ *xrs2*∆an^R^ | This Study |
| WXY2479 | DMA with *mms2*∆::nat^R^ *xrs2*∆an^R^ | This Study |
| DBY747 | *MAT***a** *his3-*∆1 *leu2-3,112 trp1-289 ura3-52* | D. Botstein |
| WXY2379 | DBY747 with *mre11*∆::*HIS3* | This Study |
| WXY2384 | DBY747 with *pol30-K164R* | This Study |
| WXY2389 | DBY747 with *mre11*∆::*HIS3 pol30-K164R* | This Study |
| WXY2390 | DBY747 with *mre11*∆::*HIS3 rev3*∆*::LEU2* | This Study |
| WXY667 | DBY747 with *rev3*∆*::hisG-URA3-hisG* | This Study |
| WXY2917 | DBY747 with *exo1*∆::*LEU2* | This Study |
| WXY644 | DBY747 with *mms2*∆::*URA3* | Lab Stock |
| WXY2394 | DBY747 with *sae2*∆::*LEU2* | This Study |
| WXY2379 | DBY747 with *mre11*∆::*HIS3* | This Study |
| WXY2918 | DBY747 with *mms2*∆::*URA3 exo1*∆::*LEU2* | This Study |
| WXY2991 | DBY747 with *exo1*∆::*LEU2 rev3*∆::*hisG-URA3-hisG* | This Study |
| HK578-10A | *MAT***a** *ade2-1 can1-100 his3-11,15 leu2-3,112 trp1-1 ura3-1* | H. Klein |
| HK578-10D | *MAT*α *ade2-1 can1-100 his3-11,15 leu2-3,112 trp1-1 ura3-1* | H. Klein |
| WXY994 | 10A *pol30-K164R* | Lab Stock |
| WXY930 | 10D *rad18*∆::*LEU2* | Lab Stock |
| WXY919 | 10A *ade2-1 ade3*∆::*hisG mre11*∆::*HIS3* | Lab Stock |
| WXY989 | 10D *pol30*∆::*HIS3*/YCpL-Pol30/pGBT-*RAD18*/YEp*RAD6* | This Study |
| WXY990 | 10D *pol30*∆::*HIS3*/YCpL-*pol30-K164R/*pGBT-*RAD18/*YEp*RAD6* | This Study |
| WXY2959 | 10A *siz1*∆::*HIS3* | This Study |
| WXY2995 | 10A *mre11*∆::*HIS3 siz1*∆::*LEU2* | This Study |
| WXY2994 | 10A *siz1*∆::*HIS3 rad51*∆::*LEU2* | This Study |
| WXY2962 | 10A *siz1*∆::*HIS3 sae2*∆::*LEU2* | This Study |
| WXY2963 | 10A *siz1*∆::*HIS3 exo1*∆::*URA3* | This Study |
| WXY2960 | 10A *mms2*∆::*URA3 siz1*∆::*HIS3* | This Study |
| WXY2975 | 10A *sae2*∆::*LEU2* | This Study |
| WXY3007 | 10A *pol30-K164R sae2*∆::*LEU2* | This Study |
| WXY3008 | 10D *rad18*∆::*TRP1 sae2*∆::*LEU2* | This Study |
| JC437 | W303 Mre11-Myc::KanMX | Lab stock |
| JC2604 | W303 Rad18-HA::*TRP1* | This Study |
| JC2605 | W303 Rad18-HA::*TRP1*, Mre11-MYC::KanMX | This Study |

**Figure S1**. Gradient plate assay showing that the nuclease activity of Sae2 plays a role in PRR. Single and double mutants were transformed with plasmids carrying wild type, the nuclease/helicase-dead mutations or the vector alone. Overnight cell cultures were imprinted on YPD or YPD + MMS at desired concentrations and incubated at 30°C for 2 days before being photographed. Strains used were isogenic to BY4741.

**Figure S2**. Control experimental data to confirm anti-PCNA antibody and detection of PCNA ubiquitination. Overnight cultures were subcultured and allowed to grow to a cell count of approximately 1x10^7^ cells/ml before being treated with 0.05% MMS (as indicated) for 90 minutes. Total cell extracts were obtained under denaturing conditions and analyzed by SDS-PAGE and western blot. (A) Monoubiquitinated PCNA is detected in wild-type yeast whole cell extracts without the need for His_n_-affinity purification. The PCNA ubiquitination band is slightly shifted up in the strain containing the Pol30-His_7_ allele compared to the native Pol30 allele (cf. lanes 5 and 6) further confirms that this band is PCNA modification. (B) Overexpression of Rad6 and/or Rad18 enhances detection of PCNA monoubiquitination; however, it is not required for the detection of monoubiquitination (cf. lanes 5 and 6). (C) A null mutation of *rad18* abolishes monoubiquitinated PCNA. Strains used were HK578-10A (wild-type) and its isogenic derivatives WXY994 (*pol30-K164R*) and WXY930 (*rad18*∆).

**Figure S3**. Control experiments to confirm di-ubiquitination of PCNA. (A) SUMOylated PCNA is observed in the absence of MMS treatment (lanes 1 and 3), but it is dependent on the Pol30-K164 residue (lanes 2 and 4), as well as *SIZ1* (lane 5). (B) Upon MMS treatment, the two prominent bands marked as Ub_1_ and Ub_2_ are deemed to be PCNA mono- and diubiquitinations, respectively, as they were shifted in the lane containing the Pol30-His_7_ cell extract (cf. lanes 1 and 3), and were abolished in the *pol30-K164R* mutations (lanes 2 and 4). As expected, they were not affected by deletion of *SIZ1* (lane 5) and only the diubiquitinated PCNA was abolished by the *mms2* null mutation (lane 6). Strains used were HK578-10A (wild-type) and its isogenic derivatives WXY994 (*pol30-K164R*), WXY2959 (*siz1*∆) and WXY2960 (*mms2∆* Δ*siz1*∆).


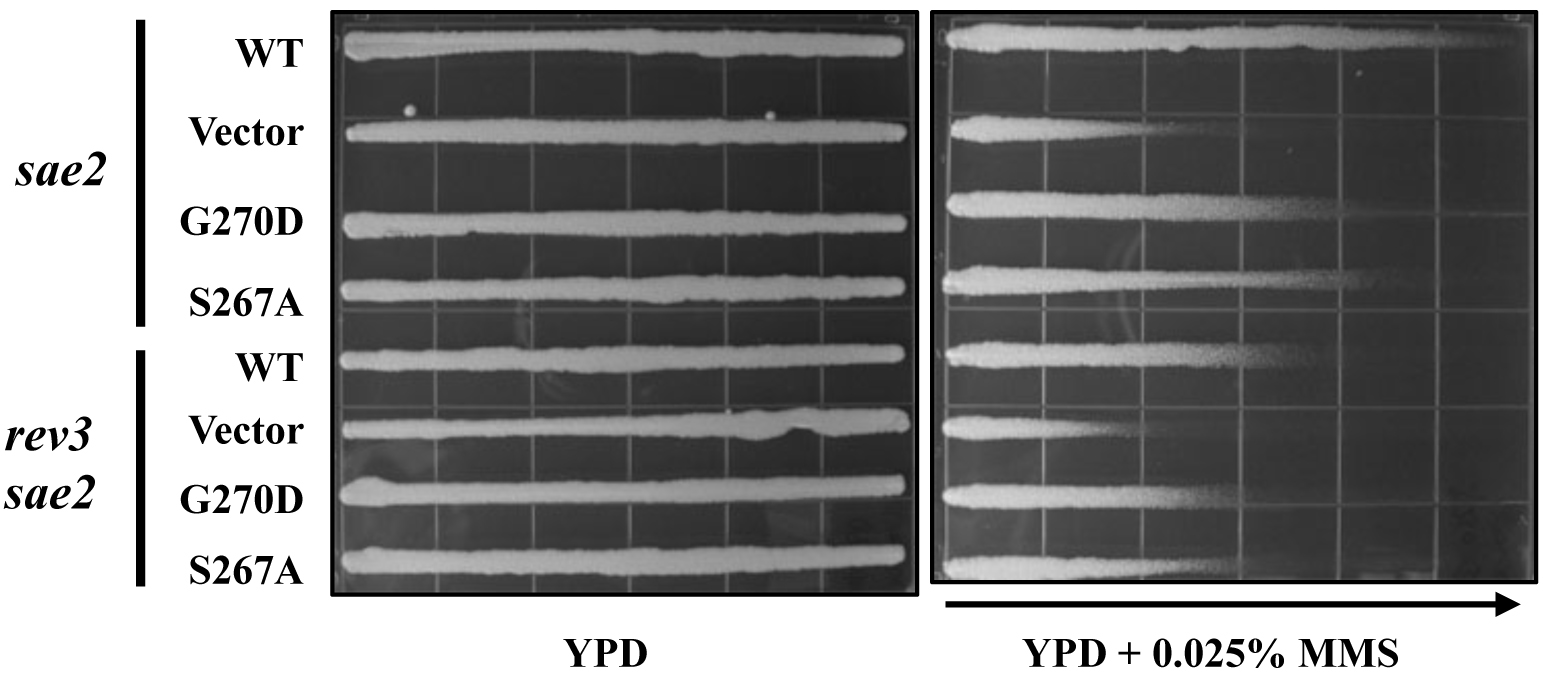

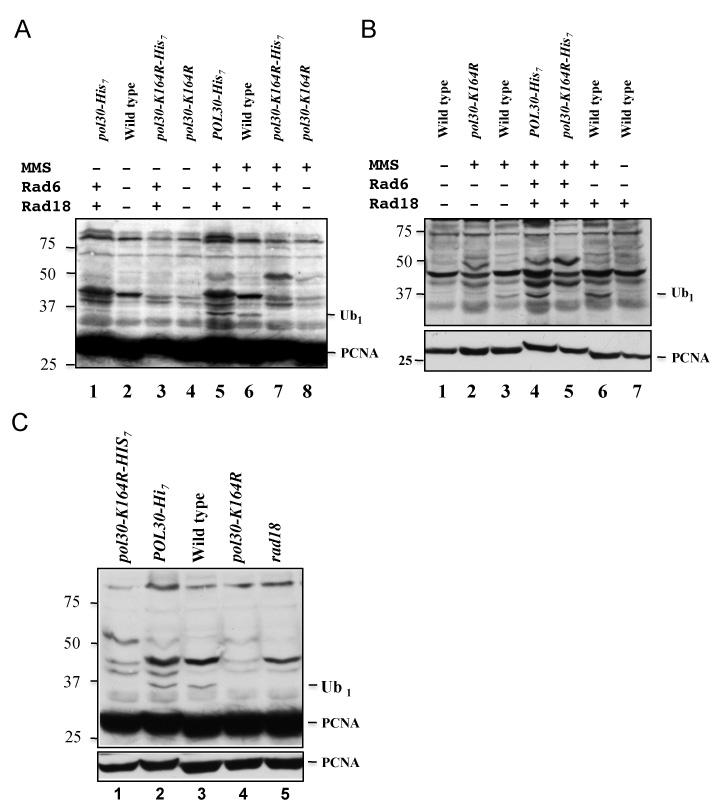


**Figure S1**


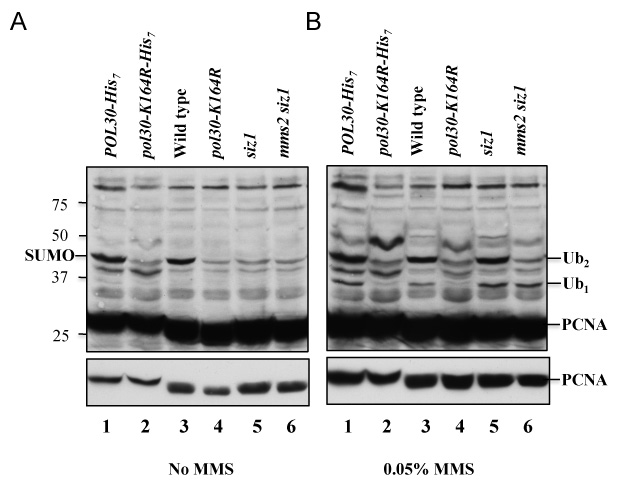


**Figure S2**

**Figure S3**
